# Prize Essays of the American Medical Association

**Published:** 1858-01

**Authors:** 


Prize Essays.—" At the meeting of the American Medical Association, held in Nashville, Tenn., in May last, the undersigned were appointed a committee to receive and examine such voluntary communications, on subjects connected with medical science, as individuals might see fit to make, and to award two prizes of one hundred dollars each to the authors of the two best essays. Notice is hereby given that all such communications must be sent, on or before the first day of April, 1858, to Grafton Tyler, M.D., Georgetown, D.C.

Each communication must be accompanied by a sealed packet containing the name of the author, which will not be opened unless the accompanying communication be deemed worthy of a prize. Unsuccessful papers will be returned on application to the Committee at any time after the first day of June, 1858; and the successful ones, it is understood, will be published in the *Transactions* of the Association.

GRAFTON TYLER, M.D.
J. C. HALL, M.D.
J. F. MAY, M.D.
THOMAS MILLER, M.D.
JOSHUA RILEY, M.D.
ALEX. J. SEMMES, M.D.
W. J. C. DUHAMEL, M.D.
*Committee of Prize Essays.*

Washington, D.C., Nov. 1857.

Notice.—The title page to the first volume of this journal, which was furnished in the November number, being deficient, and the publishers having determined to alter the style, the subscribers will find in the present number a new title page, which they are requested to substitute for the former.

The regular number of pages in each number of the Review, under its enlarged form, is One Hundred and Ninety-two, but, in consequence of a press of matter, sixteen pages extra have been added to the present number.

